# Comparative Analysis of Nutritional and Bioactive Properties of Aerial Parts of Snake Gourd (*Trichosanthes cucumerina *Linn.)

**DOI:** 10.1155/2016/8501637

**Published:** 2016-11-22

**Authors:** Ruvini Liyanage, Harshani Nadeeshani, Chathuni Jayathilake, Rizliya Visvanathan, Swarna Wimalasiri

**Affiliations:** ^1^Division of Nutritional Biochemistry, National Institute of Fundamental Studies, Hantana Road, Kandy, Sri Lanka; ^2^Department of Food Science and Technology, Faculty of Agriculture, University of Peradeniya, Peradeniya, Sri Lanka

## Abstract

The present investigation was carried out to determine the nutritional and functional properties of* T. cucumerina*. Water extracts of freeze dried flowers, fruits, and leaves of* T*.* cucumerina* were evaluated for their total phenolic content (TPC), total flavonoid content (TFC), antioxidant activity, *α*-amylase inhibitory activity, and fiber and mineral contents. Antioxidant activity, TPC, and TFC were significantly higher (*P* ≤ 0.05) in leaves than in flowers and fruits. A significant linear correlation was observed between the TPC, TFC, and antioxidant activities of plant extracts. Although, leaves and flower samples showed a significantly higher (*P* ≤ 0.05) amylase inhibitory activity than the fruit samples, the overall amylase inhibition was low in all three parts of* T. cucumerina*. Soluble and insoluble dietary fiber contents were significantly higher (*P* ≤ 0.05) in fruits than in flowers and leaves. Ca and K contents were significantly higher (*P* ≤ 0.05) in leaf followed by fruit and flower and Mg, Fe, and Zn contents were significantly higher (*P* ≤ 0.05) in leaves followed by flowers and fruits. In conclusion,* T. cucumerina* can be considered as a nourishing food commodity which possesses high nutritional and functional benefits for human health.

## 1. Introduction

*Trichosanthes cucumerina* is a well-known plant, the fruit of which is mainly consumed as a vegetable. The plant is commonly called snake gourd, viper gourd, snake tomato, or long tomato in many countries. It is an annual climber belonging to the family Cucurbitaceae and commonly grown in Asian countries including Sri Lanka, India, Malaysia, Peninsula, and Philippines [1]. The fruit is usually consumed as a vegetable due to its high nutritional value. The plant is a rich source of functional constituents other than its basic nutrients such as flavonoids, carotenoids, phenolic acids, and soluble and insoluble dietary fibers and essential minerals, which makes the plant pharmacologically and therapeutically active [2, 3]. The plant contains proteins, fat, fiber, carbohydrates, minerals, and vitamins A and E in high levels. The predominant mineral elements are potassium (121.6 mg/100 g) and phosphorus (135 mg/100 g) and also sodium, magnesium, and zinc are found in fairly high amounts [4].

In ancient medicine* T. cucumerina* was used for treating headache, alopecia, fever, abdominal tumors, bilious, boils, acute colic diarrhea, haematuria, and skin allergy. It has a prominent place in medicinal systems like* Ayurveda* and* Siddha *[2]. The whole plant including roots, leaves, fruits, and seeds is reported to show medicinal properties such as antidiabetic, antibacterial, anti-inflammatory, anthelmintic, antifebrile, gastroprotective, and antioxidant activity [1, 2, 5–7]. In Sri Lanka, it is the aerial parts of* T. cucumerina *that are used in the traditional medicinal system for treating disease conditions.* T. cucumerina* is one of the major ingredients in several polyherbal preparations that are prescribed in Sri Lanka for the control of Diabetes Mellitus [1, 6]. In a study done by Arawwawala et al. [1], the hot water extract of aerial parts of* T. cucumerina *was found to significantly reduce the blood glucose levels and improve the glucose tolerance of normoglycemic and STZ-induced diabetic rats. In addition to this, Arawwawala et al. have done several studies on Sri Lankan* T. cucumerina *and have reported it to show antioxidant [8], anti-inflammatory [9], antimicrobial [10], and gastroprotective [11] properties. However, to date, there are no detailed studies on the micronutrient content and functional properties of aerial parts of Sri Lankan* T. cucumerina. *Therefore, the present study was conducted to determine the antioxidant potential, total phenolic and total flavonoid content, *α*-amylase inhibitory activity, soluble and insoluble dietary fiber content, and the essential mineral element content of* T. cucumerina *grown in Sri Lanka.

## 2. Material and Methods

### 2.1. Plant Samples

Fresh snake gourd fruits, leaves, and flowers belonging to the variety TA-2 were collected from Horticultural Crop Research and Development Institute (HORDI), Gannoruwa, Sri Lanka, in September 2014. Fruits and leaves were harvested three months after cultivation while fruits were collected two weeks after fruit set. All the chemicals and solvents used were of analytical grade.

### 2.2. Preparation of Plant Extracts for Antioxidant, Total Phenolic, and Total Flavonoid Assays

Nearly 50 g of fresh and disease-free snake gourd leaves, flowers, and fruits were washed with distilled water and sliced. The pieces were freeze-dried, ground into fine powder, and stored at −20°C till further analysis. Freeze dried samples (0.1 g) were homogenized at 5000 rpm for 10 minutes in 7.5 mL of deionized water. The homogenized plant samples were centrifuged at 7000 rpm for 15 minutes in a microcentrifuge (5340R, Germany). The supernatant was filtered through Whatman® 42 filter paper. The extracts were appropriately diluted with distilled water and used for analysis.

### 2.3. Total Phenolic Content (TPC)

The total phenolic content (TPC) of the extracts was determined colorimetrically as described by Samatha et al. [12] with slight modifications. The reaction mixture was prepared by mixing 50 *μ*L of sample extract, 105 *μ*L of 10% Folin-Ciocalteu's reagent dissolved in deionized water, 80 *μ*L of sodium carbonate (Na_2_CO_3_, 7.5%, w/v), and 15 *μ*L of deionized water. After 3 minutes, Na_2_CO_3_ was added and the mixture was incubated for 30 minutes at room temperature. The absorbance was taken using UV-Visible microplate spectrophotometer (Multiskan®, 2011) at 760 nm. Results were expressed in terms of milligrams of Gallic acid equivalents (mg of GAE) per gram dry weight (g DW). All tests were conducted in triplicate.

### 2.4. Total Flavonoid Content (TFC)

The procedure described by Agbo et al. [13] was followed with slight modifications. A volume of 0.5 mL of aqueous extract was taken in to a test tube and 2 mL of distilled water was added followed by the addition of 0.15 mL of sodium nitrite (NaNO_2_, 5%, w/v) and allowed to stand for 6 min. Afterward, 0.15 mL of aluminum trichloride (AlCl_3_, 10%, w/v) was added and incubated for 6 min, followed by the addition of 2 mL of sodium hydroxide (NaOH, 4%, w/v), and the volume was made up to 5 mL with distilled water. After 15 min of incubation, absorbance was measured at 510 nm using UV-Visible microplate spectrophotometer. Results were expressed in terms of mg of catechin equivalents (CE) per gram of extract.

### 2.5. Antioxidant Capacity

#### 2.5.1. DPPH (2,2-Diphenyl-1-picrylhydrazyl Radical Scavenging Activity)

The DPPH assay was performed according to the method described by Sanjeevkumar et al. [14] with modifications. DPPH solution (100 *μ*L) was added to different volumes (0, 30, 60, 90, 120, and 150 *μ*L) of aqueous sample extract and diluted with distilled water until the volume reached 250 *μ*L and was allowed to stand for 30 minutes in dark at room temperature. The absorbance was read at 517 nm. The sample concentration providing 50% inhibition (IC_50_) was calculated.

#### 2.5.2. ABTS (2,2′-Azino-bis(3-ethylbenzothiazoline-6-sulphonic Acid)) Radical Scavenging Activity

The total antioxidant capacity of the extracts was determined using ABTS radical. The ABTS radical was generated by reacting ABTS (1.25 mM) with potassium persulphate (PP) (2.0 mM), which acts as the radical generator. From each extract 50 *μ*L was mixed with 150 *μ*L of ABTS radical solution and absorbance was read over 6 minutes at 1-minute interval at 734 nm. The radical scavenging activity after lapse of 6 minutes was calculated as percentage of ABTS discolouration [15]. Results were presented as mM of Trolox equivalents (mM of TE) per gram dry weight (g DW).

#### 2.5.3. Ferric Reducing Antioxidant Power (FRAP) Assay

The ability to reduce ferric ions was measured using the method described by Shukla et al. [16] with some modifications. The FRAP reagent was prepared by mixing 300 mM sodium acetate buffer (pH 3.6), 10.0 mM (tripyridyl triazine) TPTZ, and 20.0 mM FeCl_3_·6H_2_O solution in a ratio of 10 : 1 : 1, respectively. FRAP reagent (150 *μ*L) was added to 100 *μ*L of sample extract and the reaction mixture was incubated at 37°C for 30 min. The absorbance was measured at 593 nm. Fresh working solutions of FeSO_4_ were used for the standard curve. The results were expressed as mM Fe^2+^ equivalents per gram of sample.

### 2.6. Potent *α*-Amylase Inhibitory Activity

#### 2.6.1. Preparation of Plant Extracts for *α*-Amylase Inhibitory Assays

Freeze dried snake gourd sample (0.1 g) was homogenized at 5,000 rpm for 15 minutes in 7.5 mL of 1% Dimethyl Sulphoxide (DMSO). The homogenized plant samples were centrifuged at 7,000 rpm for 15 minutes in a microcentrifuge. The supernatant was used for enzyme assays.

#### 2.6.2. DNSA (3,5-Dinitrosalicylic Acid) *α*-Amylase Inhibitory Assay

The assay was performed according to Sudha et al. [17] with slight modifications. The assay mixture consisted of 50 *μ*L of *α*-amylase from porcine pancreas (A3176 SIGMA) in phosphate buffer (PBS) (0.02 M; 6.7 mM NaCl; pH 6.9) and 50 *μ*L plant extracts in different concentrations ranging from 5 to 25 mg/mL. The content was incubated for 30 minutes at room temperature followed by the addition of 100 *μ*L of 1% soluble starch (S2004 SIGMA) and incubated for another 3 minutes at room temperature. The reaction was terminated by adding 100 *μ*L DNSA color reagent and placed in 85°C water bath for 15 minutes. After cooling, the sample mixture was diluted with 900 *μ*L of distilled water and absorbance was measured at 540 nm. The control samples were prepared without plant extracts. The % inhibition was calculated and the results were expressed in terms of IC_50_ value. Acarbose was used as the standard inhibitor.

#### 2.6.3. Starch-Iodine *α*-Amylase Inhibitory Assay

Starch-iodine assay was carried out as described by Sudha et al. [17] with slight modifications. Assay reaction was initiated by adding 40 *μ*L of PBS, 40 *μ*L of plant extracts in different concentrations (5–25 mg/mL), and 40 *μ*L of enzyme in PBS into microplate wells. After 10 minutes of incubation at 37°C, 40 *μ*L of 0.3% soluble starch solution was added and again incubated for another 15 minutes at 37°C. To stop the reaction 20 *μ*L of 1 M HCl was added, followed by the addition of 100 *μ*L of iodine solution (5 mM I_2_ and 5 mM KI). Immediately after addition the absorbance was taken at 620 nm.

#### 2.6.4. Starch Hydrolase *α*-Amylase Inhibitory Assay

The inhibitory activities of plant extracts were quantified based on turbidity measurements according to previous work done by Liu et al. [18] with slight modifications. Assay was initiated by adding 100 *μ*L of PBS, 40 *μ*L of enzyme solution, and 40 *μ*L of plant inhibitor solution. The reaction mixture was incubated for 10 minutes at 37°C and thereafter 100 *μ*L of 1% starch solution was added and the mixture was again incubated for another 15 minutes at 37°C. The turbidity change was monitored at 660 nm. The % inhibition was calculated.

### 2.7. Determination of Mineral Content by Microwave Assisted Digestion

This analysis was performed according to the method described by Negi et al. [19] with slight modifications. From each freeze dried plant sample, 0.2 g was digested with 5 mL of HNO_3_ (69%) and 1 mL of H_2_O_2_ (30%) in a microwave digestion system and diluted to 50 mL with deionized distilled water. A blank digest was carried out in the same way. The digestion condition in microwave digestion unit was programmed as 15 min ramping time, 10 min holding time or digestion time at 180°C, and 15 min cooling time. Calibration curves were prepared for each mineral using 1000 ppm stock solutions of relevant standard (Fe, Zn, Ca, Mg, and K). Necessary dilutions were done in order to get absorbance values. Mineral concentration was given as mg/100 g fresh weight (FW).

### 2.8. Determination of Insoluble and Soluble Dietary Fiber

This analysis was performed according to the method described by Shin [20] with minor modifications. Duplicates of 1 g sample were weighed and suspended in phosphate buffer (0.08 M) and digested sequentially with heat-stable *α*-amylase (A3306 Sigma) (pH 6; 100°C; 30 min), protease (P3910 Sigma) (pH 7.5; 60°C; 30 min), and amyloglucosidase (A9913 Sigma) (pH 4–4.6; 60°C; 30 min) to remove protein and starch. Enzyme digestates were filtered through glass fritted crucibles. Crucibles containing insoluble dietary fiber were rinsed with dilute ethanol followed by acetone and dried overnight in a 105°C oven and weighed to nearest 0.1 mg. Filtrates plus washings were mixed with 4 volumes of 95% ethanol to precipitate materials that were soluble in the digestates. After 1 h, precipitates were filtered through fritted crucibles. One of each set of duplicate insoluble fiber residues and soluble fiber residues was ashed in a muffle furnace at 525°C for 5 h. Another set of residues were used to determine protein as Kjeldahl nitrogen. Soluble or insoluble dietary fiber residues were obtained through calculation. Total dietary fiber was calculated as the sum of soluble and insoluble dietary fiber.

### 2.9. Statistical Analysis

Data were analyzed using the SAS statistical software version 9.1.3 (SAS Institute Inc., Cary, NC). Results were calculated and expressed as mean ± standard deviation (SD) of 3 independent analyses. *P* values of ≤0.05 were considered to be significant.

## 3. Results 

### 3.1. Total Phenolic and Total Flavonoid Content

Table 1 lists the total phenolic content and the total flavonoid content of* T. cucumerina *aerial parts analyzed in the present study. The differences between the extracts for total phenolic content were significant (*P* ≤ 0.05). Leaf samples of* T. cucumerina *showed the highest phenolic content whereas the fruit samples showed the least. As in TPC values, TFC values also differ in the same manner in aerial parts of* T. cucumerina *(Table 1). Leaf samples of* T. cucumerina *showed the highest flavonoid content and the fruit samples showed the lowest phenolic content.

### 3.2. Antioxidant Capacity

**Figure 1:**
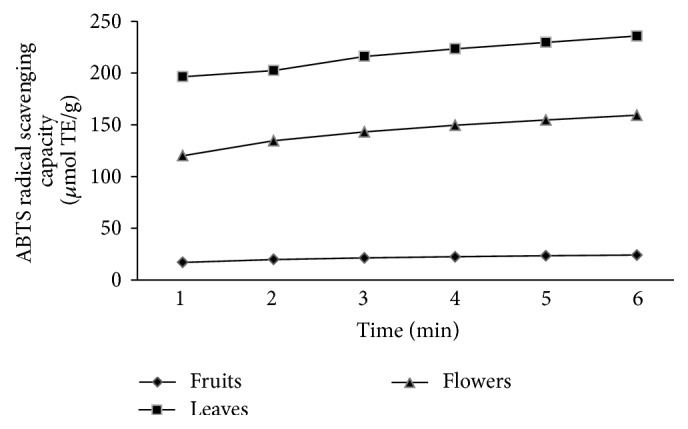
Changing of radical scavenging activity of ABTS assay with time.

In the DPPH assay (Table 1), leaves sample had the lowest IC_50_ value which indicates that it possessed the highest antioxidant activity (*P* ≤ 0.05) among all three samples. Fruit extract showed the least significant antioxidant activity (*P* ≤ 0.05) by giving the highest IC_50_ value as 10.83 ± 0.7 mg/mL. According to the ABTS assay results (Table 1), leaves had significantly higher (*P* ≤ 0.05) antioxidant activity followed by the flower and fruit extract (Figure 1). The antioxidant data in FRAP assay also vary in the same manner. Leaves samples of* T. cucumerina *showed the highest antioxidant activity while the fruit samples showed the least activity.

According to statistical data, the antioxidant activity of* T. cucumerina *was in accordance with their amount of total phenolic and flavonoid contents (Table 2). Total phenolic and flavonoid contents showed strong positive correlation with the ABTS and FRAP assay values and negative correlation with IC_50_ values of DPPH assay.

### 3.3. Potent *α*-Amylase Inhibitory Activity

**Figure 2:**
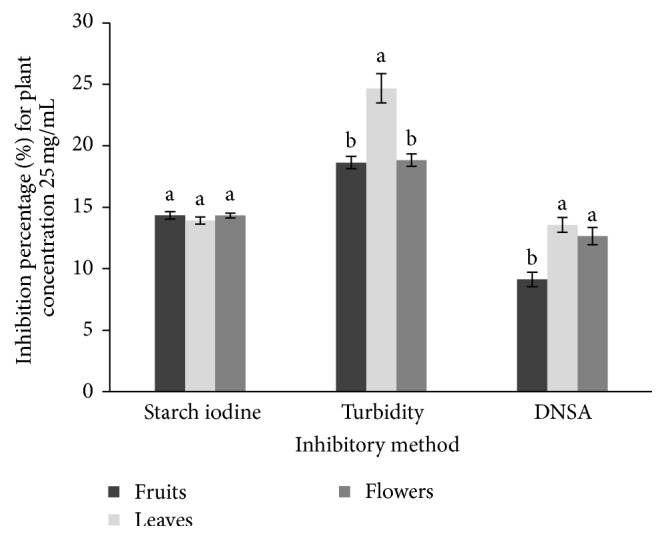
Potent *α*-amylase inhibitory activity values for different assays. Means followed by the same letter(s) (a and b) within a cluster are not significantly different at *P* > 0.05.

Results indicate that the aerial parts of* T. cucumerina *possess *α*-amylase inhibitory activity but not in significantly high level. According to the three amylase inhibitory assays, none of the parts of* T. cucumerina *gave an IC_50_ value within the concentration of 25 mg/mL (Figure 2). Acarbose was used as the positive control (Table 3).

### 3.4. Mineral Element Content

Potassium was recorded as the highest (*P* ≤ 0.05) mineral element followed by calcium and magnesium. Iron and zinc were recorded as the least. Among all three parts, flower samples had the highest mineral element content while the lowest was recorded in the fruit samples, except for calcium and potassium (*P* ≤ 0.05). On the contrary, fruit samples were significantly high in potassium (*P* ≤ 0.05) (Table 4).

### 3.5. Insoluble and Soluble Dietary Fiber

Insoluble dietary fiber (IDF) content of all three parts was higher than soluble dietary fiber (SDF). When considering the total dietary fiber (TDF) content, fruit samples had significantly higher fiber content (31.76 ± 3.7 g/100 g) followed by flowers and leaves (21.63 ± 1.71 g/100 g and 9.95 ± 1.25 g/100 g, resp.) (Table 5). The statistical variation was the same as in TDF for both SDF and IDF.

## 4. Discussion

Snake gourd contains a rich variety of nutrients, vitamins, and minerals that are essential for human health, including significant levels of dietary fiber, a small amount of calories, and high levels of protein. These medicinal plants are reported to possess different pharmacological properties due to the presence of phytochemicals such as alkaloids, flavonoids, glycosides, tannins, and steroids. It is well-known that antioxidative properties of plants help to protect the body from free radical damage in cell structures, nucleic acids, lipids, proteins, and other body components and thereby reduce the risk and progression of many acute and chronic diseases like cancer, cardiovascular diseases, diabetes, and other metabolic syndromes [21]. The results of the present study on screening the phenolic and flavonoid contents and also the total antioxidant capacity confirmed that all three parts of* T. cucumerina *possess antioxidative properties. These results are in agreement with those reported by Choudhary et al. [22] in India, where the leaves possessed the highest total phenolic content while fruits possessed the lowest. As in TPC values, TFC values also differ in the same manner in aerial parts of* T. cucumerina*. According to Ademosun et al. [23], the total flavonoid content of* T. cucumerina* was significantly (*P* ≤ 0.05) higher than that of a tomato variety of* Lycopersicon esculentum *Mill. var.* esculentum*.

Antioxidants are substances that have the ability to neutralize free radicals. In a normal healthy human body, the scavenging of prooxidants such as reactive oxygen species (ROS) and reactive nitrogen species (RNS) is effectively regulated by various antioxidant defense systems. With the exposure to adverse physicochemical, environmental, or pathological conditions, the regularly maintained balance is shifted in favor of prooxidants. This results in oxidative stress which is highly responsible for adverse health conditions including autoimmune destruction in the human body [24]. Hence, assessing the antioxidant capacities in various foods or herbs is important to elevate the antioxidant levels and their action in the body. In the present study, the antioxidant capacity was measured using DPPH, ABTS, and FRAP assays and all three extracts were found to show potential antioxidant activity. In a study done by Choudhary et al. [22], the DPPH radical scavenging activity was higher for the leaf extract than the fruit extract. In another study, Singh and Prakash [25] reported the IC_50_ values for leaves and fruit as 278.29 ± 2.98 *μ*g/mL and 260.30 ± 2.23 *μ*g/mL, respectively. The antioxidant activities of* T. cucumerina *well correlated with the amount of total phenolic and flavonoid contents. Several reports have shown a close relationship between total phenolic content and antioxidant activities [22].

Natural plants or their products are related in retarding the absorption of glucose by inhibiting starch hydrolyzing enzymes, such as pancreatic amylase and glucosidase. The inhibition of these enzymes delays carbohydrate digestion thereby reducing the glucose releasing rate and consequently lowers the increase in postprandial plasma glucose. Several indigenous medicinal plants have a high potential in inhibiting *α*-amylase activity [26]. Though the present findings did not give significant enzyme inhibition activity for aerial parts of* T. cucumerina*, the findings of Ademosun et al. [23] have shown a higher inhibitory effect on *α*-amylase activity with an IC_50_ value of 2.15 ± 0.09 mg/mL.

Dietary minerals and trace elements are chemical substances required by living organisms in addition to carbon, hydrogen, nitrogen, and oxygen that are present in nearly all organic molecules. These elements perform various functions, including building of bones and cell structures, regulating the body's pH, carrying charge, and driving chemical reactions. Minerals and trace elements are usually obtained through the diet. Though food sources of animal origin provide most essential minerals, some plants are also considered as rich sources of minerals. Several other researchers have reported positive results for mineral element analysis of* T. cucumerina*. According to Ilelaboye and Pikuda [27],* T. cucumerina* seeds contain iron (187 mg/kg DM), zinc (37.5 mg/kg DM), calcium (1643 mg/kg DM), magnesium (1896 mg/kg DM), and potassium (8704 mg/kg DM).

Most of the foods with a low or medium glycemic index are reported to contain considerable amounts of dietary fiber [28, 29]. There are two different types of fiber, soluble and insoluble. Both are important for health, digestion, and preventing diseases. The increased satiety associated with high fiber foods may result in reduction of appetite due to decreased rate of digestion and absorption [30]. According to Braaten et al. [31], among the two types of dietary fiber, the SDF fraction has shown more promising effect on lowering postprandial blood glucose values. The study done by Osuagwu and Edeoga [32] also indicated positive results for total fiber content of* T. cucumerina *leaves as 12 g/100 g similar to that of the present study. However, Hettiaratchi et al. [33] had reported that incorporating* T. cucumerina *fresh fruit into a salad meal did not increase the total fiber content in comparison to the control meal incorporated with* Lasia spinosa* rhizome.

The results of this study suggest that* T. cucumerina* is rich in phenolic compounds and has good antioxidant activity. The plant can also be considered as a good source of essential mineral elements and rich in both soluble and insoluble dietary fiber. Several studies have also proven that* T. cucumerina *possesses so many functional health benefits [1–11]. Thus,* T. cucumerina* can be considered as a natural source of functional nutrients which may yield many beneficial effects to the human body.

## 5. Conclusion

From the present study it is concluded that the water extract of* Trichosanthes cucumerina*, fruits, leaves, and flowers possessed significant amounts of beneficial compounds. The highest concentration of phenolic compounds, flavonoids, and antioxidant capacity was found in the leaves and least in the fruits. The *α*-amylase inhibitory activity of the plant was not significant. Furthermore, the mineral element content of the leaves and flowers is considerably higher than that of the fruit, while the fiber content is significantly high in the fruit compared to the leaves and flowers.

## Figures and Tables

**Table 1:** Total phenolic content and total flavonoid content and antioxidant capacity.

Plant part	TPC	TFC	DPPH	ABTS	FRAP
(Dry weight)	(mg GAE/g)	(mg CE/g)	IC_50_ (mg/mL)	(*µ*mol TE/g)	(mM Fe^2+^/g)
				6th minute	
Fruits	4.64 ± 0.3^c^	0.77 ± 0.1^c^	10.83 ± 0.7^a^	24.05 ± 0.8^c^	0.40 ± 0.01^b^
Leaves	27.39 ± 1.2^a^	6.05 ± 0.1^a^	3.08 ± 0.2^b^	235.71 ± 8.5^a^	4.24 ± 0.08^a^
Flowers	19.97 ± 1.6^b^	4.46 ± 0.1^b^	4.16 ± 0.1^b^	159.19 ± 2.4^b^	4.08 ± 0.11^a^

Means followed by the same letter within each column are not significantly different at *P* ≤ 0.05, according to the least significant difference test.

**Table 2:** Correlation of antioxidant capacity and total phenolic and total flavonoid contents.

Characteristics	TPC	TFC	DPPH	ABTS	FRAP
TPC					
TFC	0.999				
DPPH	−0.961	−0.971			
ABTS	0.998	0.995	0.945		
FRAP	0.919	0.933	0.991	0.896	

**Table 3:** Acarbose positive control for *α*-amylase inhibitory assays.

Inhibitory method	IC_50_ (*μ*g/mL)
Starch-iodine method	80
Turbidity method	83
DNSA method	78

**Table 4:** Mineral content by microwave assisted digestion.

Plant part	Mineral elements (mg/100 g dry weight basis)				
	Fe	Zn	Ca	Mg	K
Fruits	4.81 ± 0.09^c^	1.21 ± 0.05^bc^	2452.31 ± 25.10^a^	1179.50 ± 17.27^c^	2472.77 ± 16.94^ab^
Leaves	11.72 ± 0.48^ab^	2.81 ± 0.35^bc^	1450.23 ± 33.25^bc^	2146.12 ± 74.88^b^	2367.89 ± 22.45^ab^
Flowers	13.87 ± 0.14^ab^	7.13 ± 0.05^a^	1351.88 ± 24.47^bc^	2533.75 ± 21.59^a^	1379.07 ± 17.10^c^

Means followed by the same letter(s) within each column are not significantly different at *P* ≤ 0.05, according to the least significant difference test.

**Table 5:** Insoluble and soluble dietary fiber.

Plant part	Insoluble dietary fiber	Soluble dietary fiber	Total dietary fiber
	(g/100 g)	(g/100 g)	(g/100 g)
Leaves	9.17 ± 0.91^c^	0.78 ± 0.02^c^	9.95 ± 1.25^c^
Flowers	17.49 ± 1.78^b^	4.14 ± 0.50^b^	21.63 ± 1.71^b^
Fruits	19.68 ± 1.50^a^	12.07 ± 1.45^a^	31.76 ± 3.70^a^

Means followed by the same letter(s) within each column are not significantly different at *P* ≤ 0.05, according to the least significant difference test.
